# Microscale dysfunction and mesoscale compensation in degenerating neuronal networks

**DOI:** 10.1162/NETN.a.552

**Published:** 2026-07-20

**Authors:** Vegard Fiskum, Nicolai Winter-Hjelm, Nicholas Christiansen, Axel Sandvig, Ioanna Sandvig

**Affiliations:** Department of Neuromedicine and Movement Science, Norwegian University of Science and Technology (NTNU), Trondheim, Norway; Department of Clinical Neuroscience, Umeå University Hospital, Umeå, Sweden; Department of Community Medicine and Rehabilitation, Umeå University, Umeå, Sweden

**Keywords:** Graph theory, Functional connectivity, Multielectrode array, ALS, Hyperexcitability, Rich club

## Abstract

Progressive neurodegenerative diseases involve neuronal dysfunction across cellular, circuit, and whole-brain levels. Despite differences in anatomical origins, vulnerable neuronal subtypes, and specific misfolded proteins, these diseases share key features. In presymptomatic phases, neural networks engage compensatory processes to maintain function, including increased centralization and reliance on a rich-club of hub nodes. While such mechanisms have supporting evidence in some disorders, they remain less established in amyotrophic lateral sclerosis (ALS), limiting understanding of potential shared presymptomatic responses. To address this, we investigated structural and functional properties of ALS patient-derived motor neuron networks compared with healthy controls using longitudinal multielectrode array recordings and graph theory-based analysis. We observed microscale dysfunction marked by TAR DNA-binding protein 43 proteinopathy, hyperactivity, and reduced spike amplitude. Structurally, ALS networks exhibited neurite hypertrophy, suggesting attempts to form new connections. Mesoscale analyses revealed functional reconfigurations, including increased rich-club connectivity and network assortativity, indicating compensatory centralization. Our findings provide novel evidence that ALS network features can be recapitulated in in vitro models, and that these networks progressively become more centralized to preserve computational capacity, imposing growing demands on hub nodes and predisposing them to further damage. These results support models proposing common network reconfiguration mechanisms across neurodegenerative diseases.

## Author Summary

This study makes significant contributions to preclinical modelling of neurodegenerative disease, with specific relevance for amyotrophic lateral sclerosis (ALS) research. By utilizing human cellular models, longitudinal extracellular electrophysiology, and advanced network analysis, we show that known features of ALS can be recapitulated in in vitro engineered neural networks, and that these networks allow for novel hypothesis testing and identification of presymptomatic pathological processes including increased centralization. This has previously been observed in other neurodegenerative diseases, but evidence has been limited in ALS. Our results advance our understanding of motor neuron network dynamics in ALS and contribute to a shared understanding of how neurodegenerative diseases affect neural networks which go beyond specific disease diagnosis, elucidating fundamental processes in neural network function and disease response.

## INTRODUCTION

Over the past decade, the role of brain network structure and function in progressive neurodegenerative disease has received increasing attention. Although different neurodegenerative diseases originate in different anatomical regions of the central nervous system and selectively affect certain vulnerable neuronal populations, there appear to be common patterns of changes in functional connectivity. These patterns include early compensatory changes in presymptomatic phases, which fail in increasingly detrimental ways as the diseases progress (Gregory et al., 2017). Even at the microscale, or cellular level, misfolded proteins and formation of aggregates are nearly ubiquitous features, although the specific proteins involved may differ, such as TAR DNA-binding protein 43 (TDP-43) in amyotrophic lateral sclerosis (ALS) (Peng, Trojanowski, & Lee, 2020). Proteinopathy is a nearly ubiquitous feature of neurodegenerative diseases, and proteins that have previously been considered distinct to certain diseases seem to interact with each other in an interactive fashion (Zhang, Kedia, & Klenerman, 2025). Indeed, TDP-43 has been found to misfold, mislocalize, aggregate, and propagate in multiple neurodegenerative diseases (Jo et al., 2020; Liao, Ma, & Dou, 2022). Understanding these shared mechanisms and responses may enable early detection, broad preventive strategies, and better treatments.

Neural networks organize their structural and functional features to facilitate high computational capacity while balancing metabolic demands and anatomical constraints, which can be characterized by graph theory (Bullmore & Sporns, 2009, 2012). We have previously outlined that well-functioning neural systems achieve this balance by developing several hallmarks of efficient computation, including scale-freeness, small-world topology, and a modular organization (Heiney et al., 2021). This organization facilitates local specialization and global integration of information processing, the latter of which is mediated by a community of interconnected high-degree hub nodes called a rich-club (Bullmore & Sporns, 2012; Griffa & Van den Heuvel, 2018). Maintaining these properties is vital for preserving network function, both at the mesoscale circuit level and at the macroscale whole-brain level, and disruptions can lead to loss of function, as seen in both traumatic brain injury and in neurodegenerative diseases.

Neural networks affected by progressive neurodegenerative disease initiate various processes to maintain network function, including changes in firing patterns (Bullmore & Sporns, 2009; Gunes et al., 2022; Pardillo-Díaz et al., 2022) and structural and functional connectivity (Pievani et al., 2014), many of which precede clinical symptoms. A promising model outlined by Hillary et al. proposes that early structural degeneration results in increased activity and functional hyperconnectivity as a short-term adaptive response (Hillary & Grafman, 2017; Hillary et al., 2015). In the longer term, these changes tend to make the networks more centralized, relying on a rich-club of hub nodes to facilitate a progressively larger fraction of information transmission (Hillary et al., 2014). While these changes may maintain function, they also place an increasing demand on a small subset of the network, which face increasing metabolic demands and become increasingly vulnerable to failure (Stam, 2024). Alongside such functional network reconfigurations, networks may also initiate other degeneration-induced responses including enhanced neurite outgrowth (Saad, Segal, & Ayali, 2015), which may contribute to maintaining network function for a time. In ALS, it has also been shown that structural network disease epicenters have altered gene expression related to metabolic processes, including adenosine triphosphate (ATP) production and mitochondrial function (Farahani et al., 2025). Findings from brain metabolic networks further indicate deviations from optimal network organization, correlating with clinical outcomes (Jin et al., 2025). Functional studies also show that ALS brain networks have increased fractions of nodes with a single connection, indicating local connectivity loss. Simultaneously, increasing degree divergence indicates mounting reliance on centralized network nodes to facilitate interconnectivity (Borgheai et al., 2020; Sorrentino et al., 2018).

Self-organized in vitro neural networks mirror the behavior of neurons and networks in the brain (Poli, Pastore, & Massobrio, 2015; Schroeter et al., 2015; Shein-Idelson, Ben-Jacob, & Hanein, 2011). Without behavioral and cognitive symptoms, graph theory can establish the network’s features consistent with high computational capacity (Heiney et al., 2021). By integrating these approaches with advanced neuroengineering and electrophysiology, we have also demonstrated that it is possible to probe micro- and mesoscale dynamic neural network reconfigurations caused by selectively induced perturbations (Weir et al., 2023), including neurodegenerative pathology (Valderhaug et al., 2020, 2021).

In this study, we investigated the microscale and mesoscale dynamics of human ALS patient induced pluripotent stem cell (iPSC)-derived motor neurons (MNs) harboring an endogenous expansion mutation in C9orf72 (ALS MNs) compared with healthy counterparts (HC MNs). We characterized the structural network qualities, assessed TDP-43 proteinopathy, and longitudinally assessed their functional connectivity by complex network analysis of extracellular electrophysiology using multielectrode arrays (MEAs). We provide evidence of activity changes and, for the first-time, demonstrate that endogenous connectivity changes in ALS patient brain networks can be recapitulated in in vitro engineered neural networks, and that these compensatory properties drive the networks toward a more vulnerable state.

## RESULTS

### ALS MNs Exhibit Endogenous TDP-43 Proteinopathy

**Figure 1.**
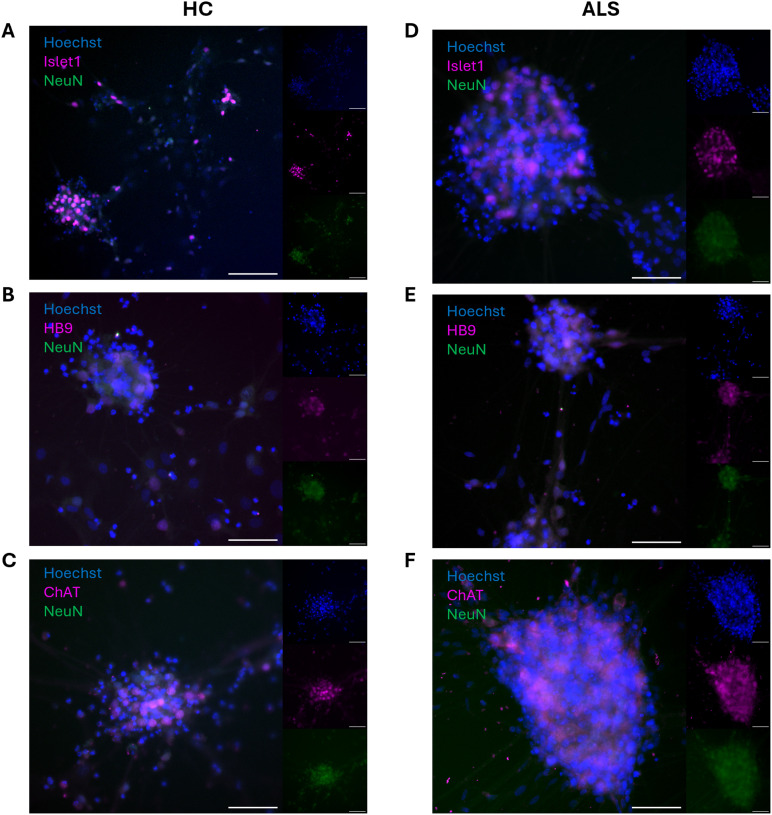
ICC confirms MN cell identity. Both healthy control (HC) and ALS patient derived MN networks showed expression of MN specific markers Islet1 (A and D), HB9 (B and E), and ChAT (C and F) alongside pan-neuronal marker NeuN. Scale bar = 100 *μ*m.

Immunocytochemistry (ICC) of HC and ALS MN networks confirmed expression of MN markers Islet1, HB9, and ChAT alongside pan-neuronal marker, NeuN, shown in Figure 1.

**Figure 2.**
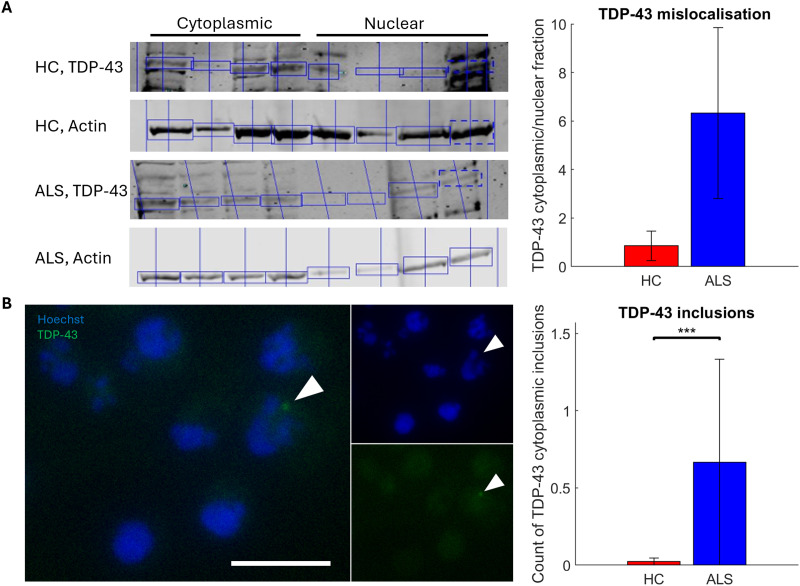
ALS MN networks exhibit TDP-43 proteinopathy. Western blot analysis of levels of TDP-43 showed that ALS MN networks had a 7.415-fold increase in the cytoplasmic to nuclear protein ratio compared with HC counterparts, *n* = 4 for HC and ALS (A). White arrows highlight inclusions of TDP-43 (green) accumulating outside of the nucleus (blue) in ALS MNs (B). Scale bar = 25 *μ*m. The number of nonnuclear TDP-43 inclusions was significantly increased in ALS MN networks compared with HC MN networks, normalized to nucleus count (*p* = 3.47 × 10^−22^), HC *n* = 419, ALS *n* = 463. *: *p* < 0.05, **: *p* < 0.01, ***: *p* < 0.001.

To assess if ALS MN networks showed signs of TDP-43 mislocalization and proteinopathy, we performed separation of cytoplasmic and nuclear cell fractions to assess the levels of TDP-43 protein in subcellular locations in HC and ALS MN networks by western blot. As shown in Figure 2A, we found a 7.415-fold increase in the fraction of cytoplasmic to nuclear TDP-43 in ALS MN networks compared with HC MN networks. Furthermore, ICC-labeling of cytoplasmic TDP-43 positive protein inclusions, as shown in Figure 2B, showed a significant increase in the number of aggregates in ALS MN networks compared with HC MN networks (*z* value = −9.6858, *p* = 3.47 × 10^−22^).

### ALS MN Networks Exhibit Microscale Dysfunction and Mesoscale Compensation

**Figure 3.**
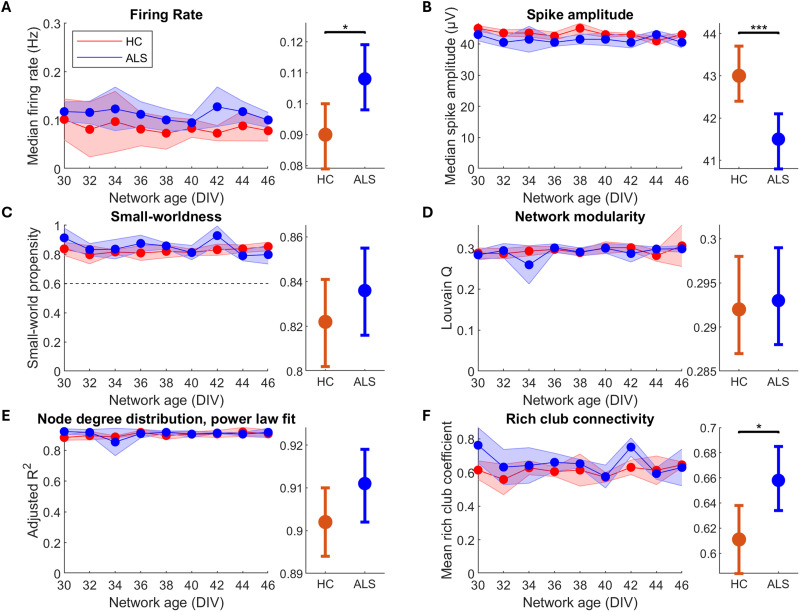
ALS patient derived and healthy MNs on HD-MEAs self-organize into networks with features of high computational capacity. Nine HD-MEA recordings per network (*n* = 4 for both HC and ALS) in the period 30–46 DIV, A–F left, were compared between HC and ALS groups using a GLMM, A–F right. Compared with HC, ALS MN networks had higher firing rate (A, *p* = 0.0140), lower spike amplitude (B, *p* = 1.09 × 10^−4^), similar small-world propensity (C, *p* = 0.315), similar modularity (D, *p* = 0.831), similar degree distributions that follow a power law, consistent with scale-free networks (E, *p* = 4.11 × 10^−3^), and increased mean rich-club coefficient (F, *p* = 0.0180). The similarity of the features shown in C–E indicate that healthy and ALS MN networks both self-organized into networks with high computational capacity. Left plots show longitudinal medians with shaded regions indicating median absolute deviation. Right plots show GLMM estimated group averages with 95% CIs. *: *p* ≤ 0.05, **: *p* < 0.01, ***: *p* < 0.001.

**Figure 4.**
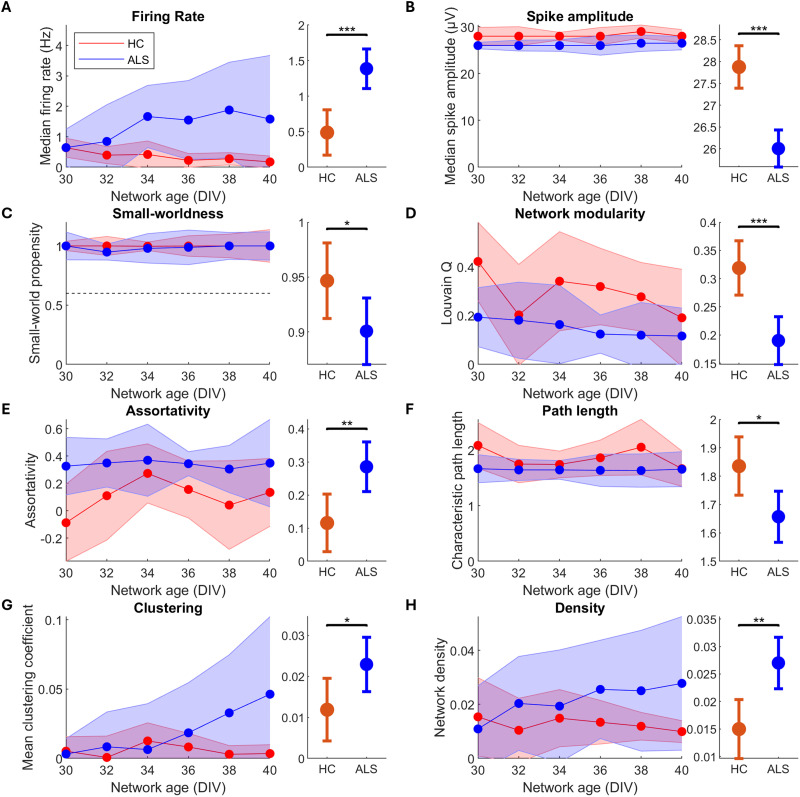
ALS patient derived MNs self-organize into networks with significant deviations from HCs on multiwell MEAs. Six multiwell MEA recordings per network (*n* = 9 for HC and *n* = 12 for ALS) in the period 30–40 DIV, A–H left, were compared between HC and ALS groups using a GLMM, A–H right. Compared with HC, ALS MN networks had higher firing rate (A, *p* = 5.01 × 10^−5^), lower spike amplitude (B, *p* = 8.07 × 10^−4^), lower small-world propensity (C, *p* = 0.0498), decreased modularity (D, *p* = 1.21 × 10^−4^), higher assortativity (E, *p* = 4.11 × 10^−3^), shorter characteristic path length (F, *p* = 0.0111), increased clustering (G, *p* = 0.0324), and increased density (H, *p* = 1.07 × 10^−3^). Left plots show longitudinal medians with shaded regions indicating median absolute deviation. Right plots show GLMM estimated group averages with 95% CIs. *: *p* ≤ 0.05, **: *p* < 0.01, ***: *p* < 0.001.

MN network activity from high-density multielectrode arrays (HD-MEAs) and six-well MEAs with 64 recording electrodes each (multiwell MEAs) is presented in Figure 3 and Figure 4 with longitudinal medians and median absolute deviations of each measurement to the left, and generalized linear mixed model (GLMM) estimated group averages with 95% confidence intervals (CIs) with statistical comparison to the right. Individual data points are shown in Supporting Information Figures S1 and S2.

MN network activity from HD-MEAs was recorded every other day from 30 to 46 days in vitro (DIV), for a total of nine repeated measurements per network, shown in Figure 3. GLMM results of activity from HD-MEAs are summarized in Table 5, with additional model details in Supporting Information Table S3. The median firing rate was significantly higher in ALS MN networks compared with HC MN networks, as seen in Figure 3A. Simultaneously, ALS MN networks exhibited significantly lower spike amplitudes compared with HC MN networks, shown in Figure 3B. Both HC and ALS networks showed spontaneously emergent properties associated with highly computationally competent networks. This included similar levels of small-worldness, as seen in Figure 3C, where all networks had small-world propensity >0.6 and similar degrees of modularity, shown in Figure 3D. The node degree distributions of the networks adhered well to a power law fitting, as seen in Figure 3E, which is consistent with scale-free networks for both HC and ALS MNs. However, to confirm scale-freeness, it is necessary to compare degree distributions to other plausible distributions (Broido & Clauset, 2019). HC and ALS MN networks were both better fitted to a power law distribution than other distributions, indicated by higher adjusted *R*^2^, shown in Supporting Information Figure S3, Supporting Information Table S1, and Supporting Information Table S2. However, goodness of fit to power law and exponential distributions could not be statistically distinguished (*p* > 0.05). Power law exponents were not significantly different between HC and ALS MN networks (*p* = 0.281, data not shown). Individual degree distributions are shown in Supporting Information Figure S4. These traits are consistent with high computational capacity as outlined in Heiney et al. (2021). Lastly, it is noteworthy that the ALS MN networks had a significantly higher rich-club coefficient compared with the HC MN networks, shown in Figure 3F. These features indicate mesoscale compensation involving increased dependency on a subset of highly interconnected nodes to maintain network computational capacity.

MN network activity from multiwell MEAs was recorded every other day from 30 to 40 DIV, for a total of six repeated measurements per network, shown in Figure 4. GLMM results from multiwell MEAs are summarized in Table 6, with additional model details in Supporting Information Table S4. Similar to HD-MEA results, ALS MN networks had significantly higher median firing rate than HC MN networks, demonstrating network hyperactivity, and significantly lower spike amplitude, shown in Figure 4B. Furthermore, the ALS MN networks had significantly reduced small-world propensity and modularity compared with HC MN networks, shown in Figures 4C and 4D, indicating a disease-associated deterioration in computational capacity. Furthermore, we found that ALS MN networks had significantly higher assortativity than HC MN networks, as well as significantly decreased path length and significantly increased clustering shown in Figures 4E, 4F, and 4G. Lastly, ALS MN networks had significantly higher density compared with HC MN networks, shown in Figure 4H.

### ALS MN Networks Exhibit Increased Outgrowth of Shorter Neurites

**Figure 5.**
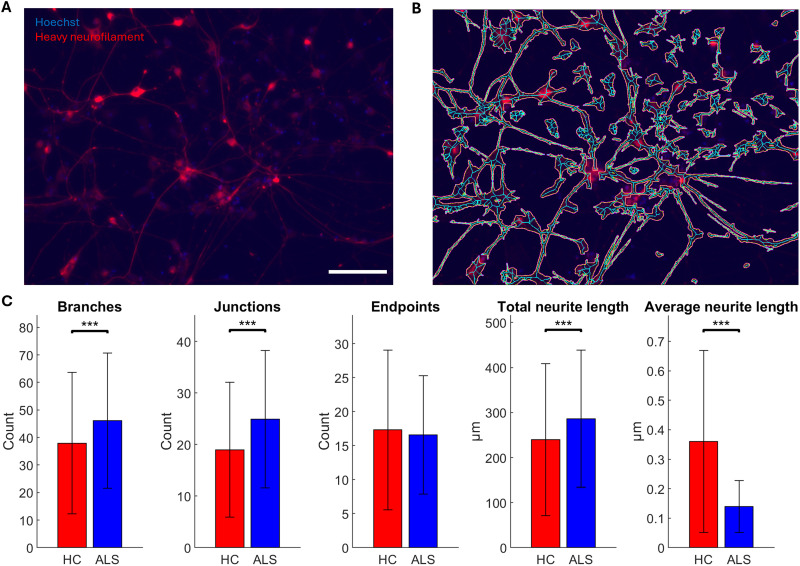
ALS MN networks have extensive structural deficits compared with HCs. MN network structure was assessed by ICC labeling of heavy neurofilament and nuclear Hoechst staining (A), using the NeuroConnectivity plugin for Fiji/ImageJ (Pani et al., 2014) to automatically identify neurites and nuclei (B). Quantification of structural features, normalized by nuclei count (C), showed that ALS MN networks had significantly more neurite branches (*p* = 6.45 × 10^−7^) and junctions (*p* = 2.29 × 10^−8^) than HC MN networks. At the same time, ALS MN networks had significantly higher total neurite length (*p* = 1.03 × 10^−4^), while the average neurite length was significantly shorter (*p* = 1.87 × 10^−13^) than in HC MN networks. The number of neurite endpoints was not significantly different (*p* = 0.0241). HC *n* = 420, ALS *n* = 558. *: *p* < 0.01, **: *p* < 0.002, ***p < 0.0002. Scale bar = 100 *μ*m.

We assessed structural features of MN networks by immunolabelled heavy neurofilament and Hoechst-labeled nuclei to determine if ALS MN networks deviated from HC MN networks. As shown in Figure 5C, ALS MN networks had significantly more neurite branches (*z* value = −4.977, *p* = 6.45 × 10^−7^) and junctions (*z* value = −5.589, *p* = 2.29 × 10^−8^), as well as significantly higher total neurite length (*z* value = −3.884, *p* = 1.03 × 10^−4^), while the average neurite length was significantly shorter (*z* value = 7.358, *p* = 1.87 × 10^−13^) compared with HC MN networks. The number of neurite endpoints was not significantly different between the two groups (*z* value = −2.256, *p* = 0.0241).

## DISCUSSION

In this study, we demonstrate for the first time that iPSC-derived neural networks from a patient with confirmed neurodegenerative disease show endogenous pathological changes in functional connectivity. Across both HD-MEAs and multiwell MEAs, ALS MNs demonstrated microscale dysfunction, involving both hyperactivity, shown in Figure 3A and Figure 4A, and decreased spike amplitude, shown in Figure 3B and Figure 4B, compared with HC MNs. Neuronal hyperexcitability is a widely reported feature of ALS (Ronchi et al., 2021; Sommer et al., 2022; Wainger et al., 2014) and other neurodegenerative diseases (Chen, Chehade, & Chu, 2025; Pardillo-Díaz et al., 2022; Targa Dias Anastacio, Matosin, & Ooi, 2022). Furthermore, the significantly lower spike amplitude in ALS MN networks, shown in Figure 3B and Figure 4B, is consistent with previous observations in ALS patient-derived MN networks (Sommer et al., 2022). Lower spike amplitude reduces Ca^2+^ influx (Scarnati et al., 2020) and may represent an adaptation of ALS MN networks to minimize Ca^2+^-induced excitotoxicity and oxidative stress. However, this also reduces the amount of neurotransmitter released (Scarnati et al., 2020) and may contribute to disruption of network function. Overall, at the microscale, we found that ALS MN networks generate more, but weaker spikes, consistent with higher metabolic cost and increased oxidative stress.

Complex network analysis showed that ALS MN networks had multiple deviations from HC networks that indicate mesoscale compensation, including increased rich-club coefficient, shown in Figure 3F, and assortativity, shown in Figure 4E. This suggests that ALS MN networks become more centralized, and especially interconnected among hub nodes, resulting in more vulnerable networks. When local connections are impaired, either by loss of nodes or loss of signal fidelity as seen in the reduced spike amplitude in our findings, networks adapt by reorganizing their functional connectivity. Because high-degree hub nodes have high connectivity and activity, they are probabilistically more available for these adaptations, which therefore leads to an increased amount of information routing through hubs (Hillary et al., 2014; Roy et al., 2017). In an early phase of this adaptation, or if the network damage is acute and requires finite reconfiguration, this may be beneficial for maintaining network communication (Hillary & Grafman, 2017). However, such reconfiguration increases the metabolic demand placed on hub nodes, and ongoing network damage such as in neurodegenerative disease leads to a continuous mounting stress on these nodes, which already represent some of the most metabolically costly components of the network (Bullmore & Sporns, 2012; van den Heuvel et al., 2012). Eventually, the nodes succumb to this burden, and since so much of the network’s overall communication is dependent on them, the network fails to maintain its function without them (Stam, 2014). While supporting evidence for such a pattern of damage and compensation has been found in cases of traumatic brain injury and Alzheimer’s disease (Hillary et al., 2015), evidence for this has been limited in ALS pathology until now. Previous work by Sorrentino et al. has shown that ALS patient brain networks become more centralized as the disease progresses (Sorrentino et al., 2018). We expand on this by showing increased rich-club connectivity in ALS MN networks on HD-MEAs. Our findings of increased network assortativity in multiwell MEAs furthermore indicate a shift toward a stricter hierarchy of node degree within the ALS MN networks. This is in line with both EEG (Iyer et al., 2015) and fMRI (Fekete et al., 2013) results from ALS patients. Networks with greater assortativity are more likely to have a highly interconnected rich-club (Rubinov & Sporns, 2010), thus supporting our findings in HD-MEA networks and providing clear evidence of pathological dynamics of centralization in ALS MN networks.

Furthermore, we found increased network density in ALS, indicating a compensatory response to maintain network function by establishing new connections. While we did find significant decreases in ALS MN network small-worldness and modularity in multiwell MEAs, shown in Figure 4C–D, these findings were not replicated in HD-MEAs, shown in Figure 3C–D. While small-worldness was significantly lower in ALS MN networks on multiwell MEAs compared with HC MN networks, shown in Figure 4C, these networks still had small-world propensity >0.6, indicating that both groups exhibited small-world features (Muldoon, Bridgeford, & Bassett, 2016). The difference in modularity outcomes between the HD-MEAs and multiwell MEAs may be due to the differences in spatial sampling, which has been shown to affect measurements of local and network properties (Maccione et al., 2010). The MEA models used in this study also differ in electrode size, and these different spatial resolutions may further contribute to the observed differences in maximized modularity (Sporns & Betzel, 2016). The observed differences across MEA platforms could suggest that ALS modules are smaller and more fragmented, and therefore more easily detected in HD-MEAs with higher spatial resolution, leading to higher maximized modularity scores. This would be in line with brain imaging studies of ALS (Renga, 2022). Additionally, we did not observe any differences in degree distributions on HD-MEA networks, shown in Figure 3E. Together, these differences from HC MN networks, and lack thereof, indicate that the ALS MN networks were in a state of compensatory reconfiguration, where adaptive mechanisms appeared to maintain network function, with only minor detrimental effects of the underlying pathology. Our results thus provide novel findings of early functional network changes in ALS.

We demonstrated clear signs of TDP-43 proteinopathy in ALS MN networks by two separate methods. Following separation of nuclear and cytosolic cell fractions, western blot analysis found a 7.415-fold increase in the fraction of cytoplasmic to nuclear TDP-43, shown in Figure 2A. ICC labeling of TDP-43 also found a significantly higher number of nonnuclear TDP-43 inclusions, shown in Figure 2B. Cytoplasmic inclusions of TDP-43 are one of the most ubiquitous signs of MN cytopathology in ALS and frontotemporal dementia (Arai et al., 2006; Neumann et al., 2006), and is aggravated by increased oxidative stress, reduced antioxidant capacity, and mitochondrial dysfunction (Kara et al., 2023). A substantial body of evidence suggest that MNs affected by ALS operate in a state of elevated oxidative stress (Cunha-Oliveira et al., 2020), and that environmental factors that exacerbate this can contribute to risk and aggravation of the pathology (D’Amico et al., 2013). Combined, these findings clearly demonstrate TDP-43 proteinopathy and signs of cellular pathology, indicating that ALS MN networks experience elevated oxidative stress and metabolic cost.

Structural analysis found that ALS MNs exhibited increased neurite outgrowth, but proportionally diminished long-range connections, shown in Figure 5C. These changes support our functional findings, indicating ongoing network reconfigurations during neurodegenerative pathology. Indeed, the structural changes may account for our multiwell MEA findings of decreased path length and increased clustering, shown in Figure 4F–G, despite the decrease in small-worldness, shown in Figure 4C. Since small-worldness is associated with low path length and high clustering (Muldoon, Bridgeford, & Bassett, 2016), these findings seem contradictory. However, given our structural findings of increasing neurite density and shorter overall neurite length, alongside our findings of increased functional density, it is possible that numerous short-range connections skew the group average path length and clustering. Notably, small-world propensity, unlike small-world index, accounts for properties like density (Muldoon, Bridgeford, & Bassett, 2016), allowing us to capture such seemingly counterintuitive network dynamics. Increasing functional connectivity, at least partially caused by increased spiking activity, as a compensatory response to structural network damage has been observed both in acute injury and chronic disease and can result in higher clustering and shorter path length (Aswendt & Hoehn, 2023). However, findings indicate that hyperactivity, hyperconnectivity, and proteinopathy are reciprocally linked, and that the compensatory responses described above can result in mounting metabolic demand, oxidative stress, hub overload, and ultimate network failure (Hillary & Grafman, 2017).

Here, we studied ALS MNs with an endogenous expansion in *C9orf72*, distinct from Clustered Regularly Interspaced Short Palindromic Repeats (CRISPR)-induced genetic predisposition or induced pathology by misfolded protein seeds. Future studies should examine MN networks derived from ALS patients with various genetic predispositions, patients with sporadic ALS, and asymptomatic donors with ALS-associated mutations, to assess if our findings are general trends of ALS or patient group specific. Also, patients with the same genetic predisposition may not share the same network features due to other factors including epigenetic imprints. In this case, iPSC-based network models in combination with complex network analysis, and with network models developed by cell reprogramming that preserves epigenetic signatures by bypassing the stem cell stage, can contribute to personalized medical assessment (Okano & Morimoto, 2022). Lastly, the methods and principles utilized in this paper can be applied to model and assess network dysfunction in other neurodegenerative diseases and to uncover which features of neural network dynamics, if any, may be shared by multiple pathologies.

In conclusion, in this study we show, for the first time, that in vitro neural networks derived from a patient with confirmed neurodegenerative disease exhibit endogenous functional network changes that reflect changes observed in multiple pathological conditions in vivo. We provide clear evidence of microscale dysfunction and mesoscale compensation in ALS MN networks, including new evidence of increased network centralization. ALS networks also exhibit elevated cytoplasmic TDP-43 protein fraction and higher levels of cytoplasmic TDP-43 inclusions, showing that common pathological hallmarks of ALS are present within such networks. At the microscale, ALS networks exhibited hyperactivity despite weaker, less robust signaling. At the mesoscale level, the networks showed increased assortativity and connectivity within a rich-club, indicating increased centralization in the face of ongoing network damage, which has been shown in other neurodegenerative diseases but has not previously been clearly demonstrated in ALS. Taken together, our findings provide novel evidence that ALS MN networks exhibit classical ALS cytopathology and self-organize into computational competent networks, but with compensatory hyperactivity and increased centralization.

## METHODS

### Experimental Design and Statistical Analysis

Human iPSCs were obtained from a healthy donor (female, 49 years, FA0000011 RUCDR Infinite Biologics and Target ALS) and a patient donor with confirmed ALS with *C9orf72* expansion mutation (female, 64 years, FA0000003 RUCDR Infinite Biologics and Target ALS), both reprogrammed according to the Sendai virus iPSC reprogramming method. iPSCs were cultured and differentiated into MNs according to the protocol by Nijssen et al. (2019), specifically the section “Procedure: E” regarding MNs from human iPSCs, with the exception that an orbital shaker was used throughout the embryoid body phase. The composition of cell media at different stages is shown in Table 1. Cells were seeded at day 10 following the start of the MN differentiation protocol. Each HD-MEA (*n* = 4 for both HC and ALS) was seeded with 80,000 live cells, each well in the multiwell MEAs with 100,000 live cells (*n* = 9 for HC and *n* = 12 for ALS), wells in a six-well plate were seeded with 800,000 MNs (*n* = 4 for both HC and ALS), and each well in the eight-well chambered slides with 40,000 live cells.

**Table 1.** Cell media compositions during motor neuron differentiation

	Days 1–2	Days 3–9	Day 10	Days 11–12	Day 13+	Supplier	Product #
*DMEM/F12-GlutaMax*	0, 5	0, 5				Thermo Fisher	31331028
*Neurobasal*	0, 5	0, 5	1	1	1	Thermo Fisher	21103049
*N2*	0.5X	0.5X				Fisher Scientific	12013479
*B27*	0.5X	0.5X	1X	1X	1X	Thermo Fisher	17504044
*Penicillin/Streptomycin*	1X	1X	1X	1X	1X	Merck	P4333
*Y-27632*	5 *μ*M		5 *μ*M			Tocris	6053
*SB-431542*	40 *μ*M					Tocris	1614
*LDN-193189*	200 nM					Tocris	6053
*CHIR-99021*	3 *μ*M					Tocris	4423
*Retinoic acid*		200 nM	200 nM			Sigma-Aldrich	R2625
*SAG*		500 nM				Tocris	4366
*Ascorbic acid*	200 *μ*M	200 *μ*M	200 *μ*M	200 *μ*M	200 *μ*M	Sigma-Aldrich	A4403
*DAPT*			10 *μ*M	10 *μ*M		Tocris	2634
*GDNF*			10 ng/mL	10 ng/mL	10 ng/mL	Peprotech	450-10
*BDNF*			10 ng/mL	10 ng/mL	10 ng/mL	Peprotech	450-02

All cell media components were sourced according to Nijssen et al. (2019), and supplements were added to base media composed of the indicated mix of DMEM/F12-GlutaMax and Neurobasal.

To compare the number of nonnuclear TDP-43 inclusions and network structural parameters in HC and ALS MN networks, we imaged immunolabelled TDP-43, heavy neurofilament, and Hoechst nuclear staining in 20% of eight separate networks from each group (*n* = 720 images for both groups). Prior to quantification of TDP-43 inclusions we removed images with no identified nuclei or TDP-43 inclusions and normalized TDP-43 inclusion count to nuclei count for each image (final *n* = 419 images for HC and *n* = 463 images for ALS). Prior to quantification of network structural parameters, we removed images with no identified nuclei and normalized structural parameters to nuclei count for each image (final *n* = 420 images for HC and *n* = 558 images for ALS). Data point distributions of HC and ALS group levels of nonnuclear TDP-43 inclusions and network structure parameters were tested for normality by Kolmogorov–Smirnov test, and did not follow a normal distribution (MATLAB R2024a, Mathworks). Group differences were therefore assessed by Wilcoxon’s rank-sum test (MATLAB R2024a, Mathworks), with Bonferroni correction for multiple comparisons.

Differences in HC and ALS MN network longitudinal electrophysiological activity was compared by GLMMs using IBM SPSS Statistics (Version 29.0.0.0). The models used genotype (HC or ALS) as a fixed effect with various network features as targets, utilizing a linear model with repeated measurements of each network (the subjects of the model).

### Electrophysiological Recordings

We studied MN networks over time both in HD-MEAs featuring 4,096 recording electrodes (3Brain Arena), and multiwell MEAs (Axion Biosystems M384-tMEA-6B). For multiwell MEAs, network electrophysiological activity was recorded using an Axion Maestro Pro acquisition tool and AxIS Navigator software (Version 3.12.2.2). Each recording lasted 30 min at 37°C and 5% CO_2_ with a sampling rate of 12.5 kHz. Prior to starting the recording, multiwell MEAs were left to settle for 15 min. The activity of all networks was recorded every other day from Day 30 to Day 40. For HD-MEAs, network electrophysiological activity was recorded using a Biocam Duplex System (3Brain) and BrainWave 5. Each recording lasted for 15 min at 37°C with a sampling rate of 18857.72 Hz. Prior to starting the recording, HD-MEAs were left to settle for 5 min. The activity of all networks was recorded every other day from Day 30 to Day 46.

### ICC

ICC was applied to confirm MN identity after differentiation and maturation of the iPSCs, assess network structure, and to investigate the presence of cytoplasmic inclusions of TDP-43. The approach was based on the work by Richter et al. (2018). Cell media was removed, and the networks were fixed for 15 min in 3% glyoxal solution consisting of 70.1% MQ H_2_O, 19.7% ethanol (Kemetyl 200-578-6), 7.8% glyoxal (40% wt. % in H_2_O, Sigma Aldrich 128465) and 0.75% acetic acid (Merck 1.00063). Then, the networks were washed with PBS (D8662, Merck) four times. The cells were then permeabilized with 0.5% Triton-X (T8787, Merck) in PBS for 5 min. Networks were then washed three times with PBS, before adding a blocking solution of 5% goat serum (PCN5000, Fisher Scientific) in PBS for 1 hr at room temperature on an orbital shaker at 30 rpm. After aspirating the blocking solution, primary antibodies in PBS with 5% goat serum were added and left overnight on a shaker table at 4°C. The next day, primary antibodies were removed, and networks were washed four times with PBS. Secondary antibodies were then added in PBS with 5% goat serum and left on an orbital shaker at 30 rpm for 3 hr, followed by nuclear staining for 10 min. The networks were then washed four times with PBS, before being washed once with milli-q water. The duration of each washing step was 5 min. All networks were fixed at 42 DIV.

MN networks were examined for expression of Islet-1 (ab109517, 1:250, Abcam), Chat (ab178850, 1:500, Abcam), HB9 (ab221884, 1:200, Abcam), NeuN (ab279295, 1:500, Abcam), heavy neurofilament (ab4680, 1:1000, Abcam), and TDP-43 (PA5–27221, 1:500, Fisher Scientific), all visualized with the same secondary antibodies (Goat anti-mouse, ab150113, 1:500, Abcam; Goat anti-rabbit, ab175471, 1:500, Abcam; Goat anti-chicken, ab150171, 1:500, Abcam) and stained for Hoechst (62249, 1,2000, Fisher Scientific). All images were acquired using an EVOS M7000 microscope with the following light cubes: DAPI (AMEP4650), CY5 (AMEP4656), GFP (AMEP4651), and TxRed (AMEP4655) and the lens Olympus UPLSAP020x, 20x/0.75 NA (N1480500).

### Image Analysis

Five structural properties of MN networks, Neurite Branches, Junctions, Endpoints, Total and Average length, as well as identification of nuclei, were assessed using the NeuroConnectivity plugin for Fiji/ImageJ (Pani et al., 2014) using the settings in Table 2.

**Table 2.** NeuroConnectivity parameters

Nuclei parameters	Neurites parameters
Segmentation method: Thresholding	Contrast enhancement enabled
Preprocessing:	Median Filter enabled
Background subtraction enabled	Median Filter scale: 2
Local contrast enhancement enabled	Tubeness Enhancement Scale: 2
Preprocessing Method: Median	Threshold Settings:
Filter size: 8	Fixed Threshold Fine: 40
Threshold method: Fixed	Fixed Threshold Rough: 55
Fixed Threshold: 50	Object filters, Min Area Fragments: 400 *μ*m^2^
Object filters:	Search area:
Min. Circularity: 0.00	Dilation search region: 2 px
Min. Area: 10 *μ*m^2^	Dilation nuclei exclusion: 15 px
Max. Area: 500 *μ*m^2^	

We used CellProfiler Version 4.2.8 to identify TDP-43 inclusions not co-localized with nuclear labeling. First, Nuclei were identified using the Hoechst labelling by applying a Threshold, before identifying objects, using the settings in Table 3.

**Table 3.** CellProfiler parameters for nuclei detection

Threshold	IdentifyPrimaryObjects
Threshold strategy: Global	Use advanced settings: No
Threshold method: Minimum Cross-Entropy	
Threshold smoothing scale: 0.0	Typical diameter of objects, in pixel units: 15–30
Threshold correction factor: 1.15	
Lower and upper bounds on threshold: 0.0–1.0	Discard objects outside of the diameter range: Yes
Log transform before thresholding: No	Discard objects touching the border of the image: Yes

The threshold image was applied as an inverted mask to the TDP-43 labeling to isolate nonnuclear TDP-43 labeling, before applying a Threshold and identifying objects using the settings in Table 4.

**Table 4.** CellProfiler parameters for cytoplasmic TDP-43 inclusion detection

Threshold	IdentifyPrimaryObjects
Threshold strategy: Global	Use advanced settings: No
Threshold method: Otsu, Two classes	Typical diameter of objects, in pixel units: 10–20
Threshold smoothing scale: 0.0	
Threshold correction factor: 2	Discard objects outside of the diameter range: Yes
Lower and upper bounds on threshold: 0.0–1.0	
Log transform before thresholding: No	Discard objects touching the border of the image: No

**Table 5.** GLMM estimates and test results, HD-MEAs

Electrophysiological measure	HC mean	HC 95% CI	ALS mean	ALS 95% CI	*F* statistic and degrees of freedom	*p* value
Firing rate (Hz)	0.09	0.079–0.1	0.108	0.098–0.119	*F*(1, 70) = 6.384	0.0140
Spike amplitude (*μ*V)	43.0	42.4–43.7	41.5	10.8–42.1	*F*(1, 70) = 12.8	1.09 × 10^−4^
Small-world propensity	0.822	0.802–0.841	0.836	0.816–0.855	*F*(1, 70) = 1.023	0.315
Modularity, Louvain Q	0.292	0.287–0.298	0.293	0.288–0.299	*F*(1, 70) = 0.046	0.831
Degree power law fit, *R*^2^	0.902	0.894–0.910	0.911	0.902–0.919	*F*(1, 70) = 2.176	0.145
Mean rich club coefficient	0.611	0.584–0.638	0.658	0.631–0.685	*F*(1, 70) = 5.874	0.0180

**Table 6.** GLMM estimates and test results, multiwell MEAs

Electrophysiological measure	HC mean	HC 95% CI	ALS mean	ALS 95% CI	*F* statistic and degrees of freedom	*p* value
Firing rate (Hz)	0.488	0.169–0.807	1.386	1.109–1.664	*F*(1, 121) = 17.689	5.01 × 10^−5^
Spike amplitude (*μ*V)	27.9	27.4–28.4	26.0	25.6–26.4	*F*(1, 121) = 32.651	8.07 × 10^−8^
Small-world propensity	0.947	0.912–0.981	0.901	0.870–0.931	*F*(1, 120) = 3.926	0.0498
Modularity, Louvain Q	0.319	0.471–0.367	0.190	0.148–0.232	*F*(1, 120) = 15.783	1.22 × 10^−4^
Assortativity	0.116	0.0286–0.203	0.286	0.210–0.361	*F*(1, 112) = 8.585	4.11 × 10^−3^
Characteristic path length	1.84	1.73–1.94	1.66	1.57–1.75	*F*(1, 120) = 6.660	0.0111
Mean clustering coefficient	0.0119	0.00424–0.0195	0.0229	0.0163–0.0296	*F*(1, 120) = 4.687	0.0324
Network density	0.0150	0.00964–0.0203	0.0270	0.0223–0.0317	*F*(1, 120) = 11.245	1.07 × 10^−3^

### Nuclear and Cytoplasmic TDP-43 Isolation

To identify the extent to which TDP-43 mislocalized from the nucleus to the cytoplasm of ALS patient derived MNs, we utilized a Thermo Scientific NE-PER Nuclear and Cytoplasmic Extraction Kit (Thermo Fisher Scientific 78833) with Thermo Scientific Halt Protease and Phosphatase Inhibitor Cocktail, EDTA-free (Fisher Scientific 10127963), following the manufacturer’s instructions. Briefly, cells from *n* = 4 networks per group were harvested from six-well plates with trypsin–EDTA (T4049, Merck) and centrifuged at 500 g for 5 min. Cell pellets were washed by resuspension in PBS (D8662, Merck), before centrifugation at 500 g for 3 min. The PBS was removed, before addition of protease and phosphatase inhibitor supplemented cytoplasmic extraction reagent 1. Cells were vortexed at the highest setting (3,000 rpm) for 15 s and incubated for 10 min before adding cytoplasmic extraction reagent 2. Cells were vortexed for 5 s before incubating for 1 min, then centrifuged at 16,000 g for 5 min. The supernatant containing the cytoplasmic extract was collected, before suspending the remaining cell pellet in protease and phosphatase inhibitor supplemented nuclear extraction reagent. The cells were vortexed for 15 s every 10 min for 40 min, before they were centrifuged at 16,000 g for 10 min. The supernatant containing the nuclear extract was then collected. All centrifugations were performed at 4°C, and all samples, extracts, reagents, vials, and pipette tips were kept on ice throughout the process. Extracts were stored at −80°C until they were analyzed by western blot, using an antibody for TDP-43 (PA5–27221, 1:1000, Fisher Scientific), and normalizing protein levels against actin levels. The fraction of cytoplasmic to nuclear TDP-43 was then compared between HC and ALS MN networks.

### Data Analysis

For multiwell MEAs, raw data were filtered using a Butterworth high-pass filter of 200 Hz and a Butterworth low-pass filter of 3 kHz. Spikes were identified using adaptive threshold crossing with a 6 standard deviation threshold, with prespike duration of 0.84 ms and postspike duration of 2.16 ms, coincidence occurrence threshold of four electrodes and coincidence event window of 80μs. Wells with a network firing rate less than five spikes per minute or with fewer than seven active electrodes were excluded from further analysis, resulting in *n* = 9 networks for HC and *n* = 12 networks for ALS. The median firing rate was determined by the number of spikes recorded for a single electrode divided by the total recording time, then taking the median of each network. To generate network connectivity matrices of multiwell MEA networks, the Pearson correlation of electrode pairs was used to generate a weighted graph, before removing 90% of the weakest connections to prune trivial connections before calculation of network features.

For HD-MEAs, *n* = 4 for both healthy and ALS conditions, raw data were filtered using a 5th-order Butterworth high-pass filter, removing low-frequency noise (below 200 Hz). Spike detection was conducted using the precise timing spike detection algorithm (Maccione et al., 2009). The threshold was set to 8 times the standard deviation of the noise, the peak lifetime period duration to 1.5 ms and the refractory time to 1 ms. Filtering and spike detection was performed in BrainWave 5 (3Brain). The median firing rate was determined by the number of spikes recorded for a single electrode divided by the total recording time, then taking the median of all electrodes for each network. To generate the network connectivity matrix of the HD-MEA recordings, spike times were separated into 100-ms bins. Then, the co-occurrences of the binned spike times were identified, resulting in a count of the number of times spikes on different electrodes co-occur. Stronger connections will have a higher tendency to co-occur. To establish a threshold for nonspurious connectivity, randomized series of corresponding data were generated by shuffling the original data. This was repeated 10 times, and co-occurrence counts that were equal to or lower than the mean of the shuffled data were removed from the connectivity matrix. Of these connections, the 1% of strongest connections were further selected, resulting in binary connectivity matrixes with a comparable number of connections. Finally, the giant component from this was analyzed using common network metrics.

The different connectivity measures and thresholding were chosen per MEA type to best identify robust functional connections within MN networks. Pearson correlations tend to identify more links with weak or intermediate weight, which is desirable for multiwell MEA networks with up to 64 nodes, whereas co-occurrence is more selective for high weight connections. The latter is preferable for HD-MEA data with up to 4,096 nodes, to eliminate spurious connections, aided further by a strict 1% threshold for edge weights.

All graph-theoretical analyses were performed using the Brain Connectivity Toolbox (Rubinov & Sporns, 2010). Small-world propensity provides an estimate to how well each network conforms to small-world principles (i.e., high clustering and low average path length), as described in Muldoon, Bridgeford, and Bassett (2016). A network with small-world propensity above 0.6 is considered small-world. The mean rich-club coefficient measures the tendency for nodes with high degree to interconnect (McAuley, da Fontoura Costa, & Caetano, 2007). Assortativity is a measure of the degree correlation of connected network nodes, and network density is the proportion of possible edges that are present in the network. Community detection was done using CDlib (Rossetti, Milli, & Cazabet, 2019), while modularity evaluation was done using NetworkX (Hagberg et al., 2008) according to (Clauset, Newman, & Moore, 2004). Briefly, community detection was performed using the Louvain algorithm, which identifies clusters of nodes that are more densely interconnected with each other than with the rest of the network, thereby grouping nodes into distinct communities. The modularity metric was used to quantify how effectively the algorithm separated nodes into these communities (Blondel et al., 2008). The Matlab functions fit, with fitType = ‘power1’, ‘exp1’, ‘weibull’, and the function fitlm applied to the logarithm of the degree distribution, were used to assess if node degree distribution followed a power law, exponential, Weibull, or log-normal distribution respectively (Matlab 2024a, MathWorks) (Broido & Clauset, 2019). The goodness of fit to these distributions was assessed by adjusted *R*^2^ and compared for HC and ALS MN networks by repeated-measures analysis of variance with post hoc between-subject comparison with Bonferroni correction for multiple comparisons using IBM SPSS Statistics (Version 29.0.0.0).

## ACKNOWLEDGMENTS

This work was supported by the Olav Thon Foundation, ALS Norge, and Alf Harborg’s fund. Western blot analysis of nuclear and cytoplasmic cell fractions was done by Proteomics and Modomics Experimental Core, PROMEC, at NTNU and the Central Norway Regional Health Authority.

## SUPPORTING INFORMATION

Supporting information for this article is available at https://doi.org/10.1162/NETN.a.552.

## AUTHOR CONTRIBUTIONS

Vegard Fiskum: Conceptualization; Data curation; Formal analysis; Funding acquisition; Investigation; Methodology; Project administration; Visualization; Writing – original draft. Nicolai Winther-Hjelm: Methodology; Software; Writing – review & editing. Nicholas Christiansen: Formal analysis; Methodology; Software; Writing – review & editing. Axel Sandvig: Project administration; Supervision; Writing – review & editing. Ioanna Sandvig: Conceptualization; Project administration; Supervision; Writing – review & editing.

## FUNDING INFORMATION

Ioanna Sandvig, Olav Thon Stiftelsen (https://dx.doi.org/10.13039/501100021720). Vegard Fiskum, Alf Harborg’s fond. Ioanna Sandvig, Stiftelsen ALS Norge.

## Supplementary Material











## TECHNICAL TERMS

TAR-DNA binding protein 43 (TDP-43): A protein frequently misfolded and involved in the formation of protein aggregates in ALS.
Amyotrophic lateral sclerosis (ALS): A progressive neurodegenerative disease that progressively affects motor neurons of the brain and spinal cord.
C9orf72: A gene susceptible to expansion mutations which predisposes a carrier to develop ALS.
Multielectrode arrays (MEAs): Cell culture vessels with multiple electrodes for extracellular electrophysiological recordings.
Immunocytochemistry (ICC): Antibody-based technique for fluorescently labeling cellular components.
Days in vitro (DIV): Refers to the number of days since a network of neurons was established in an in vitro culture.
