# Supplementary material for: Microscale dysfunction and mesoscale compensation in degenerating neuronal networks
Source: Netw Neurosci. 2026 Jul 20;10(3):594–612. doi: 10.1162/NETN.a.552 (PMC13418254; doi:10.1162/NETN.a.552)
Supplement: Supplementary file 1 [file netn-10-3-594-s001.pdf]

Supplementary material

Supplementary figure 1, Individual data points, HD-MEAs

Individual data points reflecting the data in Figure 3, showing HC (red) and ALS (blue) MN networks.

Supplementary figure 2, Individual data points, Multiwell MEAs

Individual data points reflecting the data in Figure 4, showing HC (red) and ALS (blue) MN networks.

Supplementary figure 3, Node degree distribution fits to alternative distributions

The distribution of node degrees from motor neuron networks on HD-MEAs were tested against power law (circle), exponential (x), log-normal (*) and Weibull (diamond) distributions. Distribution fit was assessed by adjusted R^2 and compared by post hoc repeated measures ANOVA. Plots show estimated group averages with 95% confidence intervals. *: $p \leq 0.05$, **: $p < 0.01$, ***: $p < 0.001$.

Supplementary figure 4, Motor neuron network degree distributions

Motor neuron networks on HD-MEAs have node degree distributions which appear to follow a power law, i.e. linear in a log-log scale.

Supplementary tables 1-2: Node degree distribution fits were estimated for power law, exponential, log-normal and Weibull distributions, with goodness of fit estimated by adjusted R^2. Group adjusted R^2 for each distribution fit was estimated by repeated measures ANOVA and compared by post-hoc, between-subject test with Bonferroni correction for multiple comparisons.

Supplementary table 1, Healthy MN networks, estimated distribution adjusted R^2

	**Adjusted R^2**		**95% Confidence Interval**		**Post-hoc distribution comparison**		
**Measure**	Mean	Std. Error	Lower Bound	Upper Bound	Power law	Exponential	Log-normal
**Power law**	0,901	0,005	0,884	0,918	-	-	-
**Exponential**	0,894	0,011	0,861	0,928	1,00	-	-
**Log-normal**	0,092	0,014	0,048	0,136	$3{,}65\text{x}10^{-12}$	$3{,}93\ \text{x}10^{-12}$	-
**Weibull**	-0,262	0,029	-0,354	-0,170	$2.51\text{x}10^{-14}$	$2{,}69\text{x}10^{-14}$	$3{,}00\ \text{x}10^{-8}$

Supplementary table 2, ALS MN networks, estimated distribution adjusted R^2

	**Adjusted R^2**		**95% Confidence Interval**		**Post-hoc distribution comparison**		
**Measure**	Mean	Std. Error	Lower Bound	Upper Bound	Power law	Exponential	Log-normal
**Power law**	0,899	0,011	0,864	0,935	-	-	-
**Exponential**	0,891	0,017	0,836	0,946	1,00	-	-
**Log-normal**	0,121	0,002	0,116	0,126	$1{,}56\text{x}10^{-11}$	$1{,}72\text{x}10^{-11}$	-
**Weibull**	-0,329	0,014	-0,375	-0,284	$3{,}23\text{x}10^{-16}$	$3{,}50\text{x}10^{-16}$	$5{,}09\text{x}10^{-11}$

Supplementary tables 3-4: Fixed effects and coefficients for GLMM-based comparison of healthy and ALS MN networks on HD and multiwell MEAs. Model coefficients assume healthy as the null model and therefore apply to the difference between ALS and healthy MN networks.

Supplementary table 3, HD-MEA GLMM fixed effects and coefficients

	**Fixed effects**		**Fixed coefficients (ALS)**				
	F (degrees of freedom)	p-value	Coefficient	Standard error	t-value	95% confidence interval	
						Lower bound	Upper bound
**Firing rate**	6,384 (1,70)	0,014	0,019	0,007	2,527	0,004	0,034
**Spike amplitude**	12,800 (1,70)	0,001	-1,599	0,447	-3,578	-2,491	-0,708
**Small-world propensity**	1,023 (1,70)	0,315	0,014	0,014	1,012	-0,014	0,041
**Modularity**	0,046 (1,70)	0,831	0,001	0,004	0,215	-0,007	0,009
**Power law fit**	2,176 (1,70)	0,145	0,009	0,006	1,475	-0,003	0,021
**Rich club coefficient**	5,874 (1,70)	0,018	0,047	0,019	2,424	0,008	0,085

Supplementary table 4, multiwell MEA GLMM fixed effects and coefficients

	**Fixed effects**		**Fixed coefficients (ALS)**				
	F (degrees of freedom)	p-value	Coefficient	Standard error	t-value	95% confidence interval	
						Lower bound	Upper bound
**Firing rate**	17,689 (1,121)	$5{,}01 \times 10^{-5}$	0,898	0,214	4,206	0,476	1,321
**Spike amplitude**	32,651 (1,121)	$8{,}07 \times 10^{-8}$	-0,002	3,27E-04	-5,714	-0,003	-0,001
**Small-world propensity**	3,926 (1,121)	0,0498	-,046	,0232	-1,981	-,092	$-3{,}505 \times 10^{-5}$
**Modularity**	15,783 (1,121)	$1{,}22 \times 10^{-4}$	-0,129	0,032	-3,973	-0,193	-0,065
**Assortativity**	8,585 (1,121)	0,004	0,170	0,058	2,930	0,055	0,285
**Characteristic path length**	6,660 (1,121)	0,011	-0,178	0,069	-2,581	-0,315	-0,042
**Clustering coefficient**	4,687 (1,121)	0,032	0,011	0,005	2,165	0,001	0,021
**Density**	11,245 (1,121)	0,001	0,012	0,004	3,353	0,005	0,019
