# Supplementary figures and images for: Microscale dysfunction and mesoscale compensation in degenerating neuronal networks
Source: Netw Neurosci. 2026 Jul 20;10(3):594–612. doi: 10.1162/NETN.a.552 (PMC13418254; doi:10.1162/NETN.a.552)

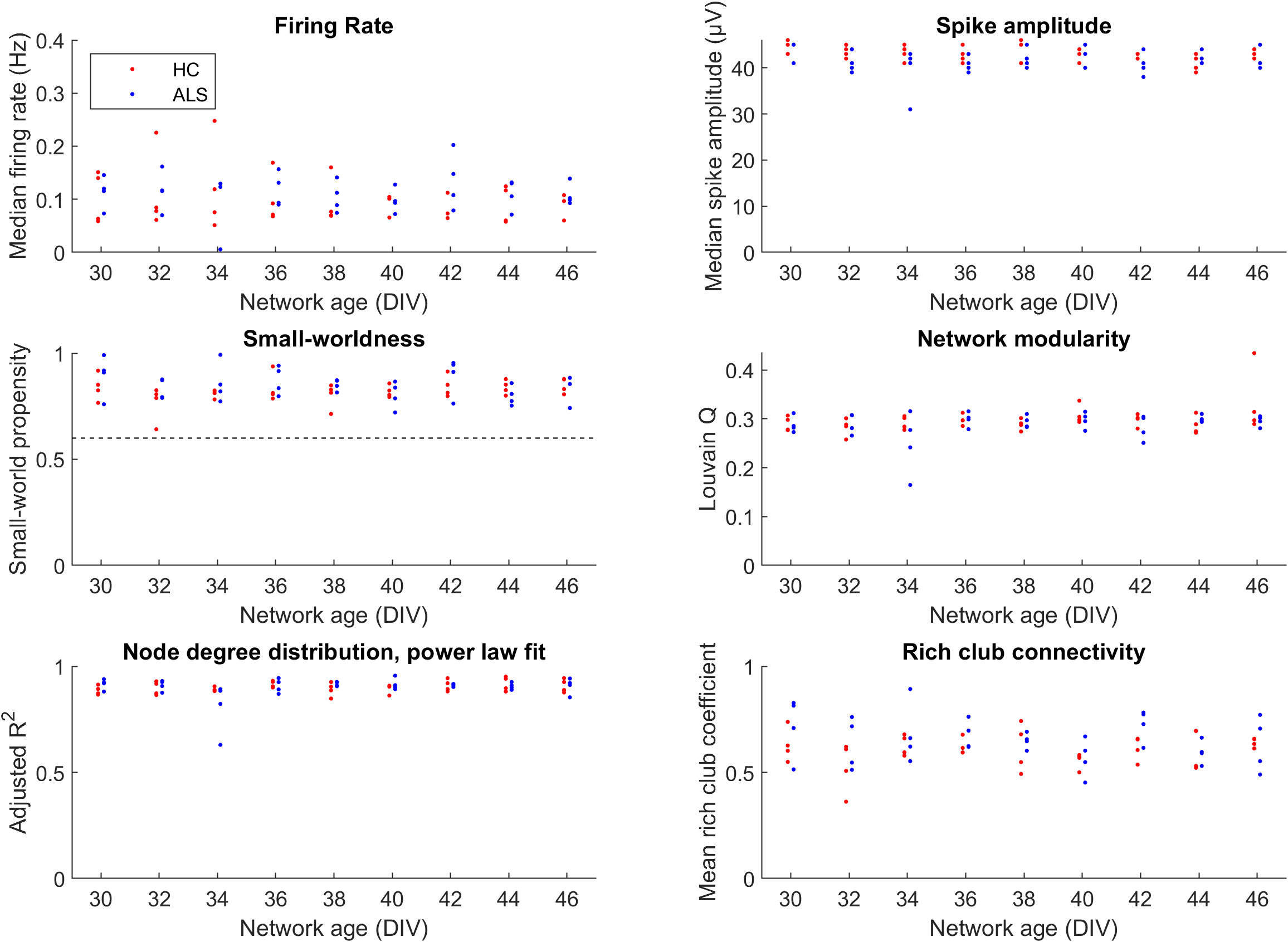

Supplement: Supplementary file 2 [file netn-10-3-594-s002.tif]

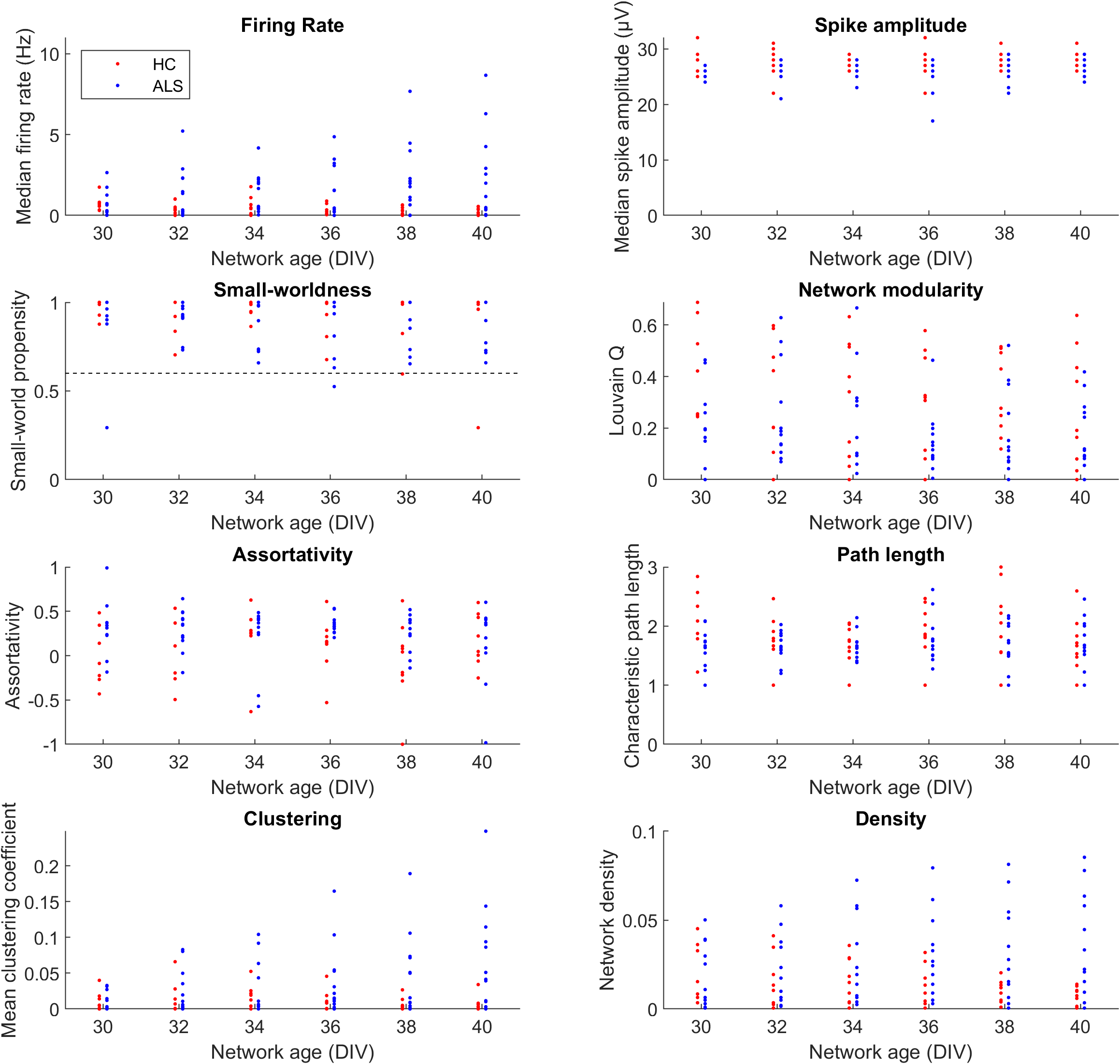

Supplement: Supplementary file 3 [file netn-10-3-594-s003.tif]

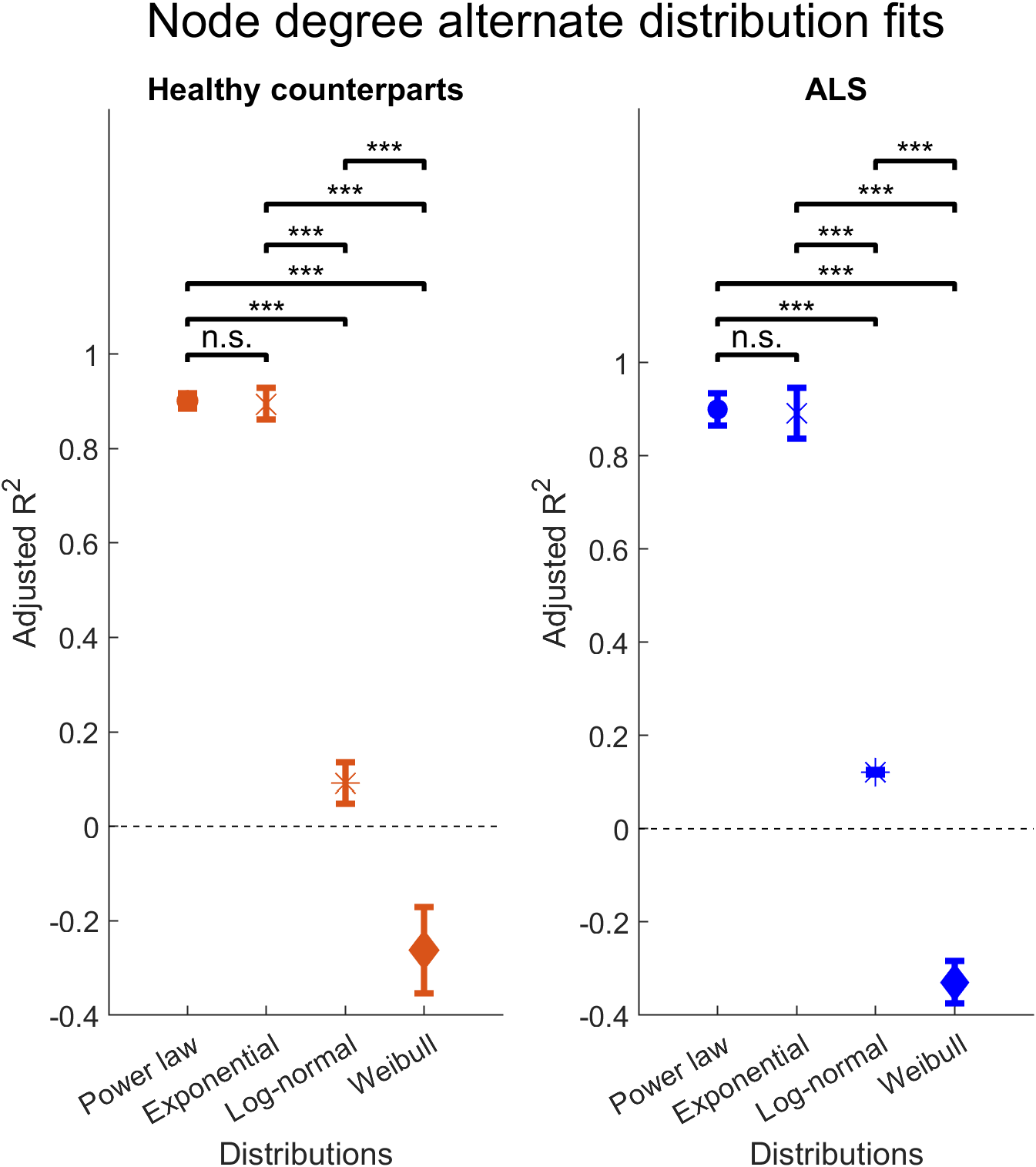

Supplement: Supplementary file 4 [file netn-10-3-594-s004.tif]

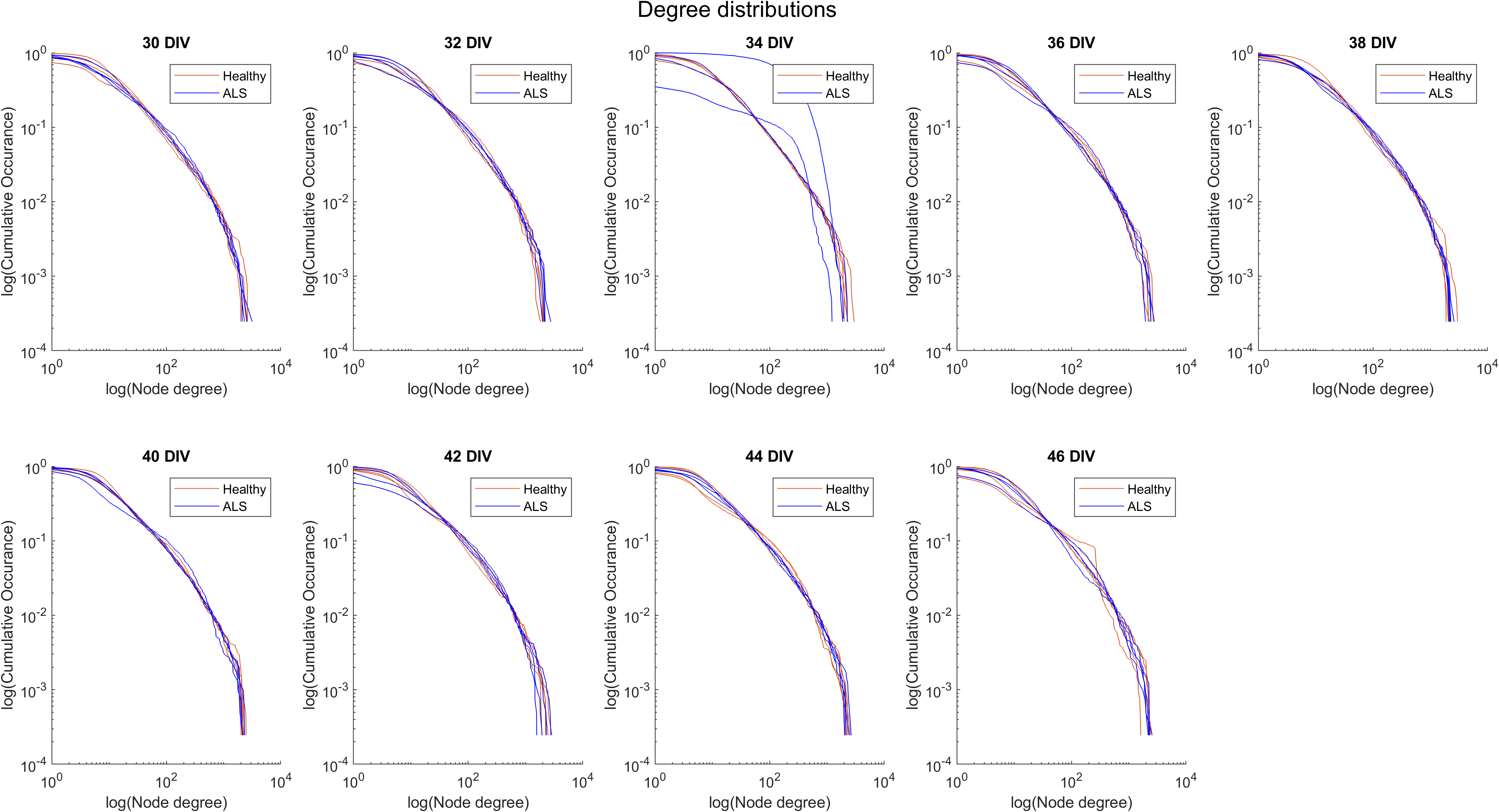

Supplement: Supplementary file 5 [file netn-10-3-594-s005.tif]
